# Fatigue Item Response among Hemoglobin-Normalized Patients with Paroxysmal Nocturnal Hemoglobinuria: PEGASUS Trial Results at 16 and 48 Weeks

**DOI:** 10.3390/jcm13061703

**Published:** 2024-03-15

**Authors:** Carolyn E. Schwartz, Katrina Borowiec, Jinny Min, Jesse Fishman

**Affiliations:** 1 DeltaQuest Foundation, Inc., Concord, MA 01742, USA; katrina.borowiec@bc.edu; 2 Departments of Medicine and Orthopaedic Surgery, Tufts University Medical School, Boston, MA 02111, USA; 3 Department of Measurement, Evaluation, Statistics, & Assessment, Boston College Lynch School of Education and Human Development, Chestnut Hill, MA 02467, USA; 4 Apellis Pharmaceuticals, Waltham, MA 02451, USA; jinny.min@apellis.com (J.M.); jesse.fishman@apellis.com (J.F.)

**Keywords:** paroxysmal nocturnal hemoglobinuria, fatigue, clinical normalization, item response

## Abstract

**Highlights:**

- Patients who switched from Ecu to Peg, reported improvements in fatigue, with greater improvements for those whose hemoglobin normalized
- We demonstrate treatment-related reductions in fatigue by highlighting the items most responsive to clinically important changes in a patient-reported outcome measure

**Abstract:**

**Background.** A common symptom of paroxysmal nocturnal hemoglobinuria (PNH) is fatigue, which in some patients can be severe. Eculizumab (Ecu) has proven efficacy in controlling intravascular hemolysis, but commonly results in persistent anemia and fatigue. Pegcetacoplan’s (Peg) efficacy was documented in the PEGASUS phase III clinical trial, showing improved hemoglobin (Hb) and patient-reported fatigue. This post-hoc analysis sought to describe this fatigue improvement related to Hb normalization using the Functional Assessment of Chronic Illness Therapy—Fatigue subscale (FACIT-F)’s individual questions to speak more directly to patients’ experience and clinicians’ day-to-day practice. **Methods.** The PEGASUS trial compared Peg with Ecu in patients who remained anemic on Ecu over 16 weeks (n = 41 and 39, for Peg and Ecu, respectively), after which all patients received Peg open label for 32 weeks (“Peg” vs. “Ecu-to-Peg” at Week 48). Hb normalization was defined as ≥12–16 g/dL for females and ≥13.6–18 g/dL for males. The FACIT-F assessed fatigue. Using the complete-case data set, Cohen’s *d* summarized the effect sizes of the mean FACIT-F item change for both study arms from the baseline to week 16 (n = 36 and 37, for Peg and Ecu, respectively) and from the baseline to week 48 (n = 30 and 29, for Peg and Ecu-to-Peg, respectively), and for Hb-normalized patients in each study arm from the baseline to week 16 (n = 14 and 0, for Peg and Ecu, respectively) and from the baseline to week 48 (n = 10 and 12, for Peg and Ecu-to-Peg, respectively). **Results.** The FACIT-F scores for both arms were worse at the baseline compared to later in the trial. Peg patients reported improvements on all fatigue items at Week 16, but Ecu patients reported improvement in only one item. At Week 48, the improvement in fatigue was maintained in Peg patients, and Ecu-to-Peg patients’ fatigue improved on all FACIT-F items. Hb normalization was achieved in 14 Peg patients but no Ecu patients at Week 16, and in 10 Peg and 12 Ecu-to-Peg patients, respectively, at week 48. The FACIT-F single items showing the largest change overall, and particularly in Hb-normalized patients across the study arms, were related to symptoms and social limitations. **Conclusions.** Peg patients reported lasting improvements in fatigue. Patients who were anemic on Ecu reported sustained improvements in fatigue with Peg treatment. Patients who had Hb normalization generally had large, clinically important improvements in fatigue items.

## 1. Introduction

Paroxysmal nocturnal hemoglobinuria (PNH) is a rare, acquired hematologic disorder with substantial morbidity and premature mortality [1]. People with PNH experience an array of symptoms including severe fatigue, which is reported in 80% of PNH patients [2]. Other common symptoms include anemia, hemoglobinuria, thrombosis, impaired kidney function, abdominal pain, dysphagia, pulmonary hypertension, chest pain, dyspnea, erectile dysfunction in males, and end organ damage [3,4,5,6,7,8,9]. PNH, marked by the dysregulation of the terminal complement pathway, leads to extravascular and intravascular hemolysis which can result in thrombosis. PNH patients generally report poor quality of life (QOL), reflecting low global-health functioning [2]. If untreated, up to 35% die within five years of diagnosis [3,4,10,11,12,13,14]. Although onset can occur at any age, the average age of diagnosis worldwide is 39.3 years (SD = 18.6) [3,4,15,16,17]. The prevalence rate is 12–38 per 1,000,000 persons, is similar across sexes, and higher among older adults [18,19]. Its clinical course is highly unpredictable [4,8].

Prior to eculizumab (Ecu), a C5 inhibitor, there were no complement treatments available for PNH. This humanized monoclonal antibody binds to C5, blocking the terminal complement pathway and thereby protecting PNH red cells from intravascular hemolysis but not extravascular hemolysis [1]. In PNH patients, intravascular and extravascular hemolysis can lead to anemia (i.e., low hemoglobin (Hb) [20,21]) resulting in symptoms, such as fatigue, abdominal pain, dysphagia, and erectile dysfunction [1]. Both anemia and fatigue have a negative effect on QOL [22], and an improvement in Hb levels is associated with a meaningful improvement in fatigue [23]. In addition, 20 to 35% of patients require transfusions, despite C5 inhibitor treatment [24]. C5 inhibitor treatment has reduced mortality and thrombosis risk, which has changed the life of these patients [1]. Ravulizumab is a second-generation anti-C5 monoclonal antibody that has a half-life four times longer than Ecu [25]. Despite C5 inhibitor treatment, some patients still experience extravascular hemolysis or C3-mediated hemolysis [26]. Hence, these patients can still remain anemic and severely fatigued [27], and require transfusions.

Pegcetacoplan (Peg) is a C3 proximal inhibitor that acts earlier in the complement cascade, thereby controlling both intravascular and extravascular hemolysis [28]. PEGASUS was a pivotal phase-three randomized controlled trial (NCT03500549) that provided evidence of the superiority of Peg versus Ecu by improving Hb in patients who were anemic on stable Ecu treatment. Hb improvement was statistically significant, indicating that its improvement was not due to chance. Changes in fatigue, as measured by the FACIT-F, were not tested due to the statistical analysis plan design that was agreed upon with regulators. Peg was also associated with clinically meaningful reductions in transfusions and the absolute reticulocyte count compared with Ecu [28].

In post-hoc analyses, clinically meaningful improvements were demonstrated in patient-reported fatigue at week 16 in Peg patients compared to Ecu patients [28,29,30,31]. The present work sought to investigate differences in patient-reported fatigue in a more detailed manner. In the present post-hoc analysis of the PEGASUS data, the aim was to transition from the previously mentioned quantitative support for enhanced fatigue (at the score level) to a more detailed characterization at the item level. This involves examining individual questions on the patient-reported outcome measure to understand how the perception of fatigue evolved during treatment, especially in patients who achieved hemoglobin normalization. Thus, the present work focuses on a practical description of the FACIT-F scale in the real-world setting for PNH.

## 2. Methods

### 2.1. Study Design

The present study is a post-hoc analysis of the PEGASUS trial data. PEGASUS is a phase III, two-arm, active-comparator controlled trial conducted across 44 sites worldwide. It compared Peg to Ecu in patients with PNH who were anemic despite prior Ecu therapy. After a 4-week run-in period with Peg plus Ecu, patients were randomized to monotherapy with Peg (n = 41) or Ecu (n = 39) for 16 weeks. Eculizumab patients were then offered the opportunity to switch to an open-label period during which they received Peg, and during which patients who received Peg during the 16-week period continued to receive Peg monotherapy. All patients (Peg and Ecu-to-Peg) were followed for an additional 32 weeks to week 48, although 3 Peg patients withdrew from the study treatment during the randomized controlled period due to treatment-emergent adverse events, and 3 Peg and 7 Ecu-to-Peg patients withdrew from the study treatment during the open-label period due to treatment-emergent adverse events (2 Peg, 7 Ecu-to-Peg) and physician’s decision (1 Peg) [30]. Eligible participants were 18 years of age or older and had hemoglobin (Hb) < 10.5 g/dL despite having received more than three months of Ecu treatment at the baseline. The detailed study inclusion and exclusion criteria have been previously described [29,32].

### 2.2. Sample

The present work focused on fatigue in PEGASUS trial participants at week 16 and at week 48. However, while in contrast to previously published primary study analyses and reporting the intent-to-treat sample results which included imputation [29,30], our analytic sample was comprised of only complete cases.

### 2.3. Measures

Clinical outcome normalization in this analysis focused on Hb normalization, which was defined as ≥12–16 g/dL for females and ≥13.6–18 g/dL for males.

**Figure 1:**
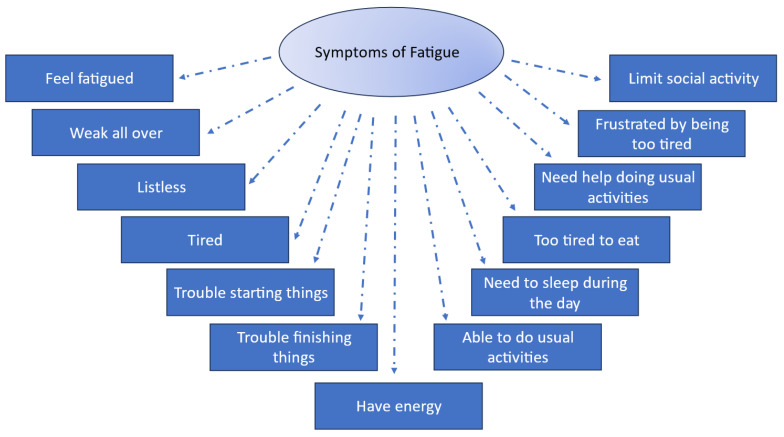
FACIT-F Items Measure Symptoms of Fatigue. FACIT items are copyrighted by David Cella, PhD, and reprinted with permission.

Fatigue was assessed using the 13-item Functional Assessment of Chronic Illness Therapy—Fatigue subscale (FACIT-F) [33]. This Likert-scaled measure asks participants to indicate the level of severity of fatigue-related symptoms (Figure 1) on a five-point scale (“not at all” to “very much”). FACIT-F scores range from 0 to 52, with most items reverse-scored so that higher scores reflect *lower* fatigue and *better* QOL.

### 2.4. Statistical Analysis

To characterize how the experience of fatigue changed over the course of treatment, descriptive statistics summarized the mean change in FACIT-F items between baseline and follow-up at weeks 16 and 48. Cohen’s *d* summarized effect sizes of the mean FACIT-F-item change for the full samples and for the Hb-normalized subsamples, and was used to identify the most responsive items by treatment arm. For the sake of interpretation, Cohen’s *d* = 0.2–0.49 is a small effect, 0.5–0.79 is a medium effect, and ≥0.8 is a large effect [34]. Items were ranked by Cohen’s *d* to reflect responsiveness to change in the overall sample and among the Hb-normalized subsample at weeks 16 and 48. A medium effect size (e.g., 0.5) is generally considered clinically important [35].

**Figure 2:**
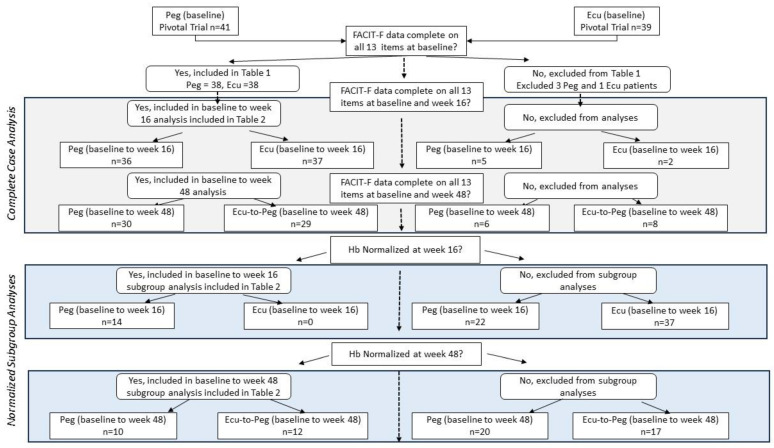
Attrition Flow Diaram.

We used a complete-case approach [36,37] in contrast with the published work that imputed missing values [29,30]. For the full sample’s complete-case analyses comparing the baseline to week 16, there were 36 and 37 patients in the Peg and Ecu study arms, respectively. From the baseline to week 48, there were 30 and 29 patients in the Peg and Ecu-to-Peg study arms, respectively. For the Hb-normalized subgroup analyses comparing the baseline to week 16, there were 14 and 0 Peg and Ecu patients, respectively. For these subgroup analyses comparing the baseline to week 48, there were 10 and 12 for Peg and Ecu-to-Peg patients, respectively. Figure 2 provides an attrition flow diagram.

Statistical analyses were implemented using IBM (Armonk, NY, USA) SPSS version 28 [38] and Microsoft 365 (Redmond, WA, USA) Excel.

## 3. Results

### 3.1. Sample

The trial arms were balanced on demographic and clinical characteristics at the baseline (Table 1). Peg patients had a mean age of 49.9 years and 66% were female. Ecu patients had a mean age of 47.7 years and 58% were female. The FACIT-F scores at the baseline were 31.1 and 31.6 for Peg vs. Ecu, respectively. Hillmen et al. [29] provide further detail on the baseline characteristics.

### 3.2. Fatigue Changes over Time

**Figure 3:**
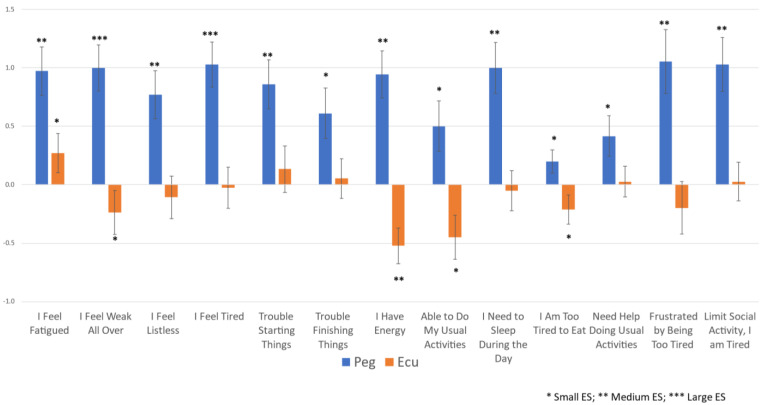
Mean FACIT-F Item Change by Treatment Arm for Overall Sample from Baseline to Week 16. FACIT items are copyrighted by David Cella, PhD, and reprinted with permission.

FACIT-F scores for Peg and Ecu were worse at the baseline compared to later in the trial. Peg patients reported improved fatigue scores on all 13 items at Week 16, whereas Ecu patients reported improvement in fatigue on one item, worse fatigue scores on four items, and no change on the other eight items (Figure 3, Table 2). Effect sizes at week 16 reflected small, medium, or large effect sizes in improvements in the Peg arm, and generally no change and small or medium effect sizes in the deterioration of those few items that changed in the Ecu arm (Figure 3, Table 2). At Week 48, Peg patients maintained their significantly better level of improvement in fatigue and showed small, medium, or large effect sizes in improvement in fatigue items compared to the baseline (Table 2). Ecu-to-Peg patients’ fatigue improved on all 13 fatigue items at week 48 compared to the baseline, reflecting small, medium, or large effect sizes (Table 2).

### 3.3. Relationship of Hb Normalization to Fatigue Changes over Time

**Figure 4:**
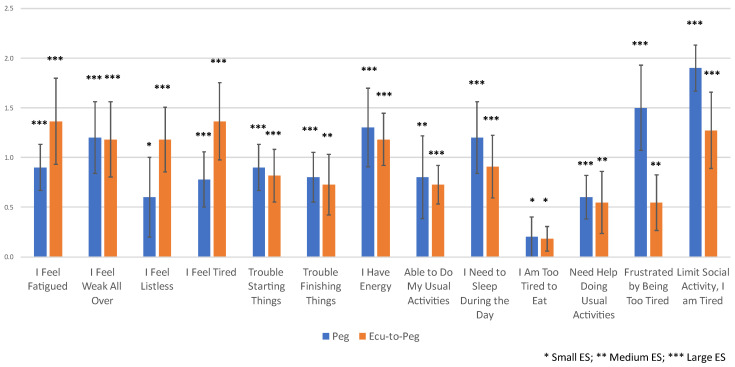
Mean FACIT-F Item Change from Baseline to Week 48 for Hb-Normalized Subgroup by Treatment Arm. FACIT items are copyrighted by David Cella, PhD, and reprinted with permission.

Hb normalization was achieved in 14 Peg patients and no Ecu patients at Week 16, and in 10 Peg and 12 Ecu-to-Peg patients at Week 48 (Table 2). Figure 4 shows the mean change from the baseline on the 13 FACIT-F items among Hb-normalized patients in both study arms at Week 48. These changes reflected predominately medium or large effect sizes for all Hb-normalized patients (Table 2, Figure 4).

### 3.4. Item Content Most Reflective of Changes in Fatigue among Hb-Normalized Patients

**Figure 5:**
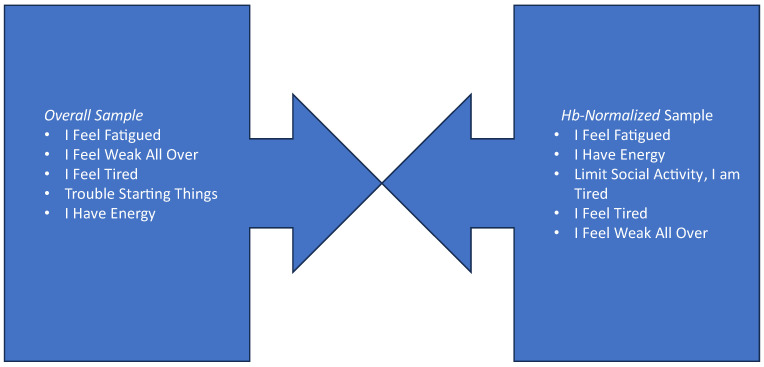
Top Five FACIT-F Items Reflecting the Most Change in Fatigue for Overall Sample and for Hb-Normalized Subgroup. FACIT items are copyrighted by David Cella, PhD, and reprinted with permission.

Table 2 also displays the ranks of the FACIT-F items on the basis of their effect sizes for the overall complete-case sample at both weeks 16 and 48, and for the Hb-normalized patients at both weeks 16 and 48. The items showing the largest change in patients across the study arms at both weeks 16 and 48 related to feeling fatigued, feeling weak all over, feeling tired, having energy, and having trouble starting things (Figure 5). Among those patients whose Hb normalized over the course of the treatment, the first four of these same items showed the largest change (Figure 5). Additionally, the item reflecting that fatigue limited social activity was also prominent (Figure 5).

## 4. Discussion

The efficacy of Peg was documented in the PEGASUS phase III clinical trial, showing the improvement of Hb, the absolute reticulocyte count, and lactate dehydrogenase, and the freedom from transfusion [29]. While patient-reported fatigue was a post-hoc outcome that showed the benefit of Peg [28], understanding the full impact of Peg treatment can be enhanced by considering patient-reported outcomes in both quantitative and qualitative ways. The present post-hoc analysis built on this past work by showing the Peg benefit on fatigue using the quantitative metric of effect size, which emphasizes clinical importance. A medium effect size is generally recognized as clinically significant [35], and the benefit of Peg reflected mostly medium or large effect sizes. The impact of Hb normalization was further emphasized in the subgroup analysis of Hb-normalized patients. By week 16, only patients in the Peg arm had achieved this Hb normalization (14 of 41) and at week 48, both treatment arms had notable numbers who achieved Hb normalization, as all patients were receiving Peg at this open-label point in the trial. The effect sizes were, on average, large.

We then illustrated how the patient’s experience of fatigue was impacted by effective treatment. Peg patients in the overall sample demonstrated large changes on FACIT-F items reflecting the feelings of fatigue—feeling fatigued or tired or weak, having energy, and trouble initiating activities. The efficacy of Peg in improving the fatigue level was further emphasized in the subgroup analysis of Hb-normalized patients. Within the Hb-normalized subgroup, the social impact of fatigue became more prominent in tandem with these same feelings of fatigue.

Putting words to feelings can be useful. While it is understood that PNH-driven anemia leads to the patient experiencing fatigue, seeing which items from a patient-reported outcome measure reflect this fatigue can speak more directly to clinicians’ day-to-day clinical practice. Further, in the patients who achieved Hb normalization, fatigue improved even more and reflected the symptom content as well as the impact of fatigue on social activity. It is our hope that this linkage between an objective outcome and the subjective experience is helpful.

### Study Limitations

The present study was limited by relatively small sample sizes, a not-uncommon context for studies of rare diseases. The analysis is limited by the post-hoc nature of this investigation, and more complex, multivariate analyses were not feasible given these relatively small samples, so we relied on more descriptive, bivariate, and illustrative approaches. The racial representation of the study sample was also somewhat limited, with a very small number of Black patients. Further, the study’s funding source and authors’ affiliations may introduce potential biases. Future research could benefit from larger, more diverse sample sizes and prospective designs to validate these findings further.

## 5. Conclusions

Peg patients reported lasting improvements in fatigue and, when patients randomized to Ecu switched to Peg during the course of the study, their fatigue levels improved greatly. Among the patients whose Hb levels normalized over the course of treatment, which was only observed in Peg patients, their fatigue improved even more notably. The item content of the most responsive items was similar in the overall sample and in the Hb-normalized subsample, reflecting improvements in the feelings of fatigue. In patients with normalized Hb, a treatment impact in terms of reduced limitations in their social role was also reported. The documented effect sizes reflect clinically important change.

## Figures and Tables

**Table 1:** Demographic and Clinical Characteristics of Patients at Baseline.

Characteristic	Pegcetacoplan (n = 38)	Eculizumab (n = 38)
Age in years: mean (SD)	49.9 (19–81)	47.7 (23–78)
Female sex: no. (%)	25 (66)	22 (58)
Race: no (%)		
Asian	5 (13)	7 (18)
Black	2 (5)	0
White	21 (55)	25 (66)
Other	0	1 (3)
Not reported	10 (26)	5 (13)
Body mass index: mean ± SD	26.7 ± 3.9	25.9 ± 4.3
Zero transfusions within previous 12 mo.: no. (%)	9 (24)	10 (26)
Median time since PNH diagnosis: no. years (range)	6.0 (1–31)	8.9 (1–38)
Hemoglobin: mean g/dL ± SD	8.80 ± 1.00	8.67 ± 0.89
FACIT-F score: mean ± SD	31.1 ± 11.1	31.6 ± 12.5

**Table 2:** Cohen’s *d* and Resulting Rank for FACIT-F Item Change by Treatment Arm, Overall and for Hb-Normalized Patients.

		All Patients										Hb-Normalized Patients							
		Baseline to Week 16					Baseline to Week 48					Baseline to Week 16				Baseline to Week 48			
FACIT-F Item No. and Content		Peg		Ecu *			Peg		Ecu-to-Peg			Peg		Ecu		Peg		Ecu-to-Peg	
		(n = 36)		(n = 37)			(n = 30)		(n = 29)			(n = 14)				(n = 10)		(n = 12)	
		*d*	Rank	*d*	Rank		*d*	Rank	*d*	Rank		*d*	Rank	*d*		*d*	Rank	*d*	Rank
1	I Feel Fatigued	0.793	4	0.266	3		0.880	5	0.683	6		1.558	2	NA		1.220	2	0.951	6
2	I Feel Weak All Over	0.860	2	−0.236	4		0.984	2	0.550	11		1.203	5	NA		1.057	5	0.945	7
3	I Feel Listless	0.635	9	−0.098	8		0.492	10	0.953	1		0.888	9	NA		0.474	12	1.096	3
4	I Feel Tired	0.894	1	−0.025	13		0.949	3	0.893	3		1.558	1	NA		0.933	9	1.060	4
5	Trouble Starting Things	0.691	7	0.134	7		0.933	4	0.933	2		0.964	8	NA		1.220	3	0.936	8
6	Trouble Finishing Things	0.472	10	0.053	9		0.537	9	0.643	7		0.559	11	NA		1.014	8	0.721	10
7	I Have Energy	0.794	3	−0.522	1		0.816	7	0.573	9		1.406	3	NA		1.039	7	1.352	1
8	Able to Do My Usual Activities	0.385	12	−0.448	2		0.446	12	0.381	13		0.334	13	NA		0.608	11	1.125	2
9	I Need to Sleep During the Day	0.764	5	−0.051	10		0.787	8	0.700	5		1.062	7	NA		1.057	6	0.870	9
10	I Am Too Tired to Eat	0.342	13	−0.212	5		0.385	13	0.573	10		0.393	12	NA		0.316	13	0.449	13
11	Need Help Doing Usual Activities	0.396	11	0.034	11		0.479	11	0.402	12		0.884	10	NA		0.858	10	0.527	12
12	Frustrated by Being Too Tired	0.644	8	−0.198	6		0.834	6	0.596	8		1.362	4	NA		1.108	4	0.584	11
13	Limit Social Activity, I am Tired	0.743	6	0.027	12		1.075	1	0.762	4		1.068	6	NA		2.575	1	1.001	5
Conditional formatting reflects magnitude and direction of Cohen’s *d* effect size (see Legend). For the sake of interpretation, Cohen’s *d* = 0.2–0.49 is a small effect, 0.5–0.79 is a medium effect, and >0.8 is a large effect. * Ranking based on absolute value of effect size. FACIT items are copyrighted by David Cella, PhD, and reprinted with permission.								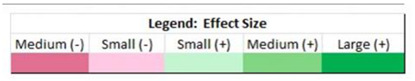											

## Data Availability

Individual participant data will not be made available. Requests for access to data from the PEGASUS trial should be addressed to Federico Grossi at federico@apellis.com. The study protocol will be available with no end date. All proposals requesting data access will need to specify how the data will be used, and all proposals will need the approval of the trial investigator team before data release.
